# Malaria among rice farming communities in Kilangali village, Kilosa district, Central Tanzania: prevalence, intensity and associated factors

**DOI:** 10.1186/s40249-017-0315-1

**Published:** 2017-07-05

**Authors:** Humphrey D. Mazigo, Susan F. Rumisha, Mercy G. Chiduo, Veneranda M. Bwana, Leonard E. G. Mboera

**Affiliations:** 1 Department of Medical Parasitology, Catholic University of Health and Allied Sciences, Mwanza, Tanzania; 2 National Institute for Medical Research, Dar es Salaam, Tanzania

**Keywords:** Malaria, Prevalence, Risk factors, Rice farming, Tanzania

## Abstract

**Background:**

Malaria remains the most important cause of morbidity and mortality in Tanzania. However, its prevalence varies from area to area depending on various ecological, socio-economic and health system factors. This study was carried out to determine malaria prevalence and associated factors among rice farming communities in the Kilangali village of Kilosa District in Central Tanzania.

**Methods:**

A cross-sectional study was conducted in May 2015, involving randomly selected persons living in the six sub-villages of the Kilangali village, namely Mlegeni, Kisiwani, Makuruwili, Kwamtunga, Upogoroni and Chamwino. A finger prick blood sample was obtained for diagnosis of malaria infection using Giemsa-stained thick smears and a rapid malaria diagnostic test. Study participants were also screened for haemoglobin levels and a total of 570 children aged ≤ 12 years of age were examined for spleen enlargement using the palpation method.

**Results:**

A total of 1154 persons were examined for malaria infection with mean age of 21.9 ± 19.69 years. The overall malaria prevalence was 14.2% and 17.5% based on microscopic examination and rapid diagnostic test, respectively. *Plasmodium falciparum* accounted for the majority (89%) of the malaria infections. The overall geometrical mean parasite density was 20.5 parasites/μL (95% *CI*: 14.6–28.8). Malaria prevalence and parasitaemia was highest among individuals living in the Mlegeni (23.9%) and Makuruwili (24.4%) sub-villages. Among the children examined for splenomegaly, 2.98% (17/570) had it. The overall prevalence of anaemia was 34.6%. Malaria infection was associated with the age groups of 1–10 years (a*OR* = 4.41, 95% *CI*: 1.96–9.93, *P* < 0.001) and 11–20 years (a*OR* = 6.68, 95% *CI*: 2.91–15.37, *P* < 0.001); and mild anaemia (a*OR* = 1.71, 95% *CI*: 1.11–2.62, *P* < 0.014) and moderate anaemia (a*OR* = 1.55, 95% *CI*: 1.01–2.39, *P* < 0.045).

**Conclusions:**

Malaria was found at the study setting and its prevalence varied according to the demographic characteristics of the study participants and between sub-villages that are closely located.

**Electronic supplementary material:**

The online version of this article (doi:10.1186/s40249-017-0315-1) contains supplementary material, which is available to authorized users.

## Multilingual abstracts

Please see Additional file 1 for translations of the abstract into the five official working languages of the United Nations.

## Background

Intensive use of malaria intervention measures has resulted in the decline of malaria transmission in some parts of the world, including in Sub-Saharan Africa [1, 2]. Recent statistics indicate that the number of malaria cases has fallen by 18% globally, from about 262 million in 2000 to 213 million in 2015. Likewise, the global number of malaria deaths has declined by 48%, from about 839 000 in 2000 to 438 000 in 2015 [3]. This decline had been reported in Tanzania [4–7], where national parasitaemia rates have also declined by almost 50%, from 17.7% in 2008 to 9.2% in 2012 [7]. Despite this remarkable achievement, however, malaria remains a major cause of morbidity and mortality in the country [7]. Malaria causes a significant burden to the health system, and is responsible for over 16 million clinical cases and over 100 000 deaths annually [3, 8].

There are variations in malaria transmission between regions of Tanzania [9, 10], with 90% of the population living in areas where malaria is highly endemic [11]. The variations in malaria transmission are likely to be explained, at least partly, by differences in socio-economic and ecological factors [8, 9, 12]. Socio-economic risk factors such as land use for agriculture, especially for rice irrigation, play an important role in the transmission of malaria due to the creation of suitable microhabitats for malaria vectors to breed [7–9]. Communities living in areas characterised by rice farming irrigation systems have repeatedly been reported to have higher entomological inoculation rates and carry the largest burden of the disease [13–15].

In Tanzania, malaria is mostly a disease affecting rural populations characterised by a poor health care system and where agriculture forms the backbone of the household economy [7]. In these areas, indirectly, malaria reduces the working capacity of the households. It is estimated that affected families are only able to use 40% of their available land for crop production compared to healthy families [16, 17]. Partly, this contributes to an increased level of poverty among rural farming communities and food insecurity [11].

In preparation for a large study, focusing on integrating the application of biolarvicides and fertiliser in rice fields to control malaria and increase rice yields in Tanzania, it was deemed necessary to generate baseline information on malaria prevalence in order to assess the effect of the intervention on malaria before and after the application of biolarvicides. Thus, the present study was conducted to determine the malaria prevalence and associated factors in the Kilangali village in Kilosa district of Central Tanzania.

## Methods

### Study area

**Fig. 1:**
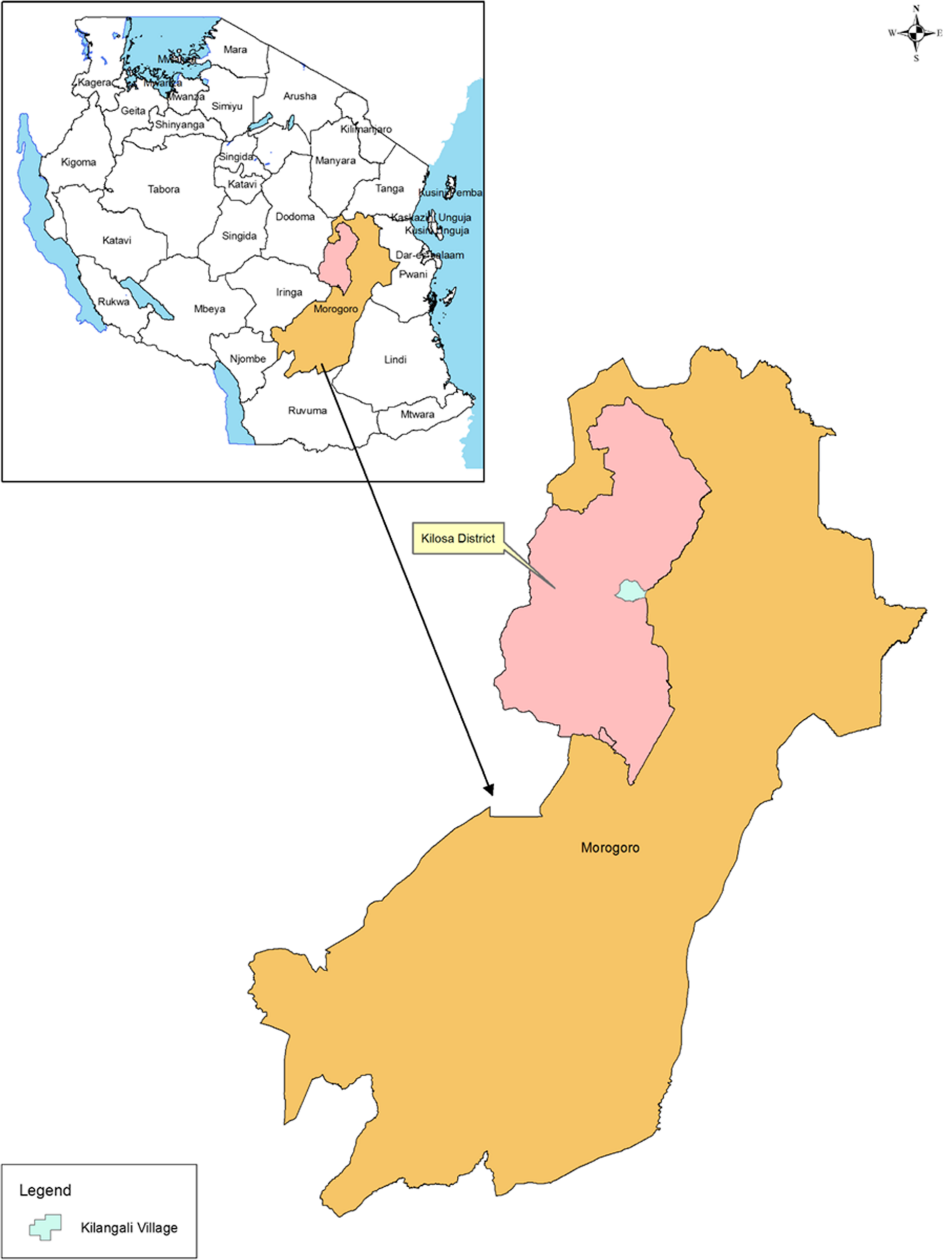
A map of Tanzania showing the location of the Kilosa district

**Fig. 2:**
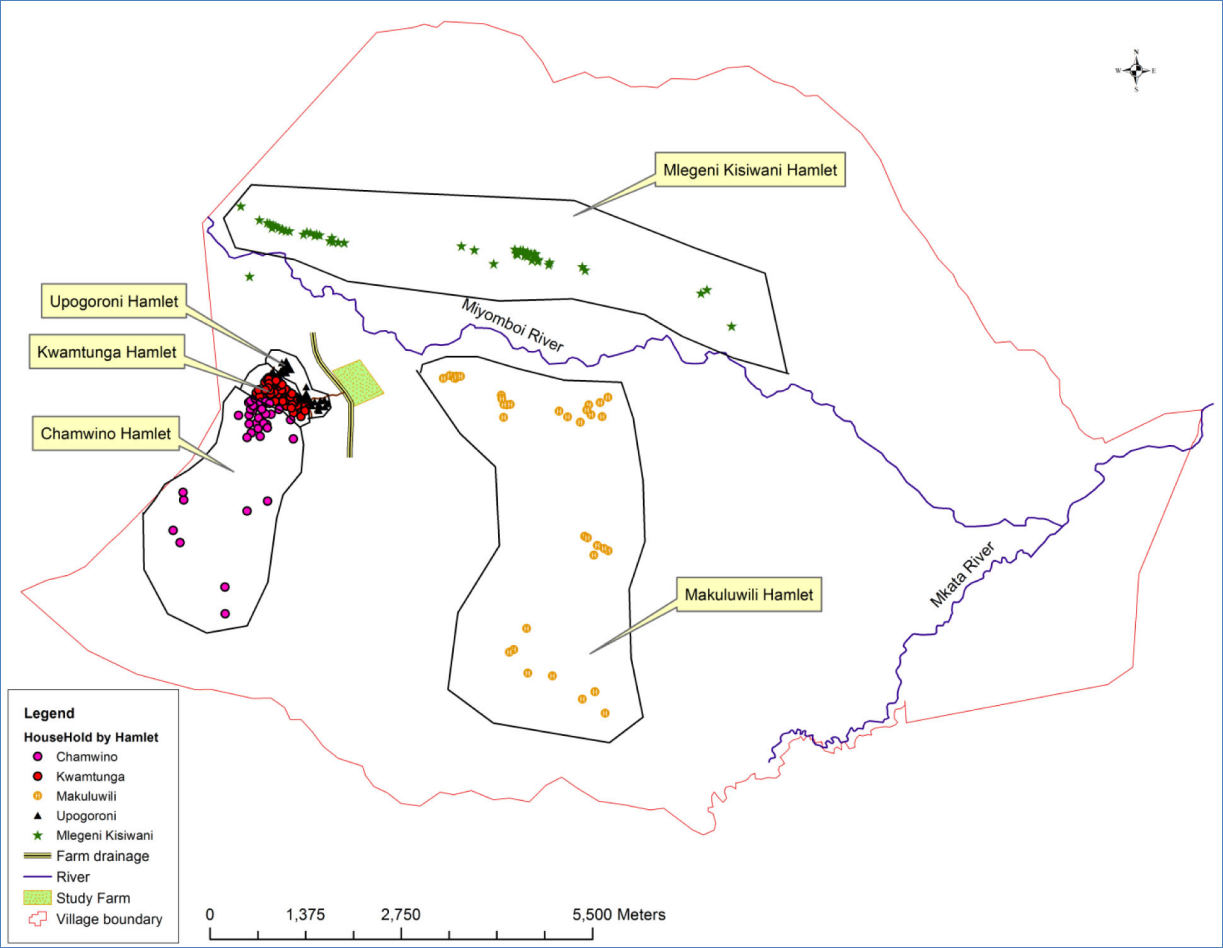
A map showing the location of the six five sub-villages of Kilangali village in relation to the rice farm where application of biolarvicides mixed with fertiliser is done

**Fig. 3:**
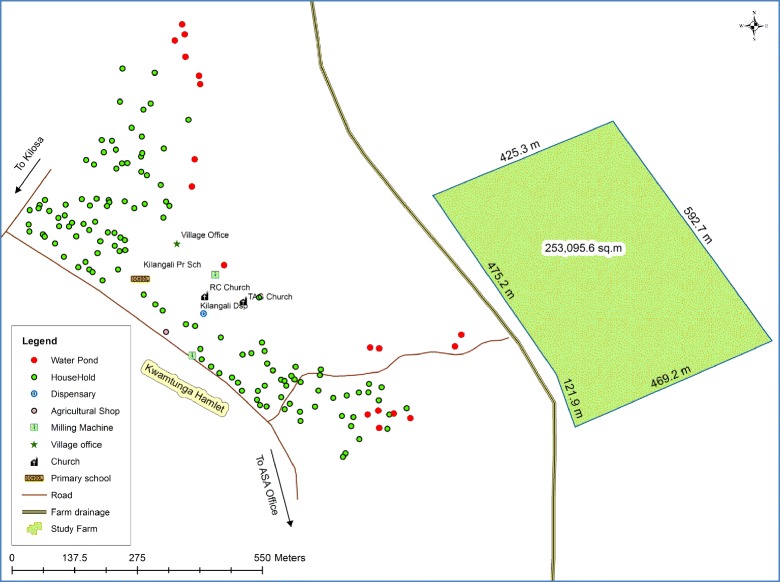
A map of the Kilangali village in Kilosa district showing the distribution of households in Upogoroni (the intervention sub-village) in relation to the rice farm where biolarvicides application mixed with fertiliser is done to control malaria transmission

This study was carried out in the Kilosa district of Central Tanzania (5°55’–7°53’ S; 36°30’–37°30’ E) (see Fig. 1) in May 2015. Kilangali village (6°58’0” S; 37°5’0” E), located in the south-eastern part of the district was selected for the study (see Fig. 2). The village population is approximately 3500 inhabitants. The area is characterised by swampy flatland and wetlands lying on the Kilangali alluvial basin. The village is bordered by the large Kilangali rice farm irrigation scheme, which totals 1200 ha. Most of the communities are small-scale rice farmers who use the traditional ground flooding irrigation practice. The selection of the Kilangali village for this study was mainly based on the fact that the type of rice farming practised by the communities provides suitable breeding sites for the major malaria vectors. Within the Kilangali village, six sub-villages were selected. These were Mlegeni, Kisiwani, Makuruwili, Kwamtunga, Upogoroni and Chamwino (see Fig. 3). Kisiwani, Mlegeni and Makuruwili are located close to the Kilangali rice seed farm irrigation scheme.

### Study design, population and sampling

This was an analytical cross-sectional survey conducted in the Kilangali village, Kilosa district, and included individuals of all age groups selected randomly from 253 out of 750 households located in the six sub-villages. Selected participants were included in the study if they had been living in the sub-villages for more than 2 years (considered as permanent inhabitants). Considering, on average, that a household has four residents [18], a sample size was approximated at 1012 participants for the entire study area. These were equally divided among the sub-villages (i.e. 84 households with about 336 individuals).

### Data collection

#### Demographic information of the study participants

Demographic information of the study participants, which included age, sex, place of residence, history of malaria treatment in the past four weeks and whether or not the participant slept under a mosquito net the night before the survey, was collected.

#### Diagnosis of malaria

School children were examined for malaria infection in their school environment and other members of the community were examined at a specified point in the sub-village. A finger prick blood sample was obtained from all study participants, following aseptic procedures. Both malaria rapid diagnostic tests (mRDTs) and microscopy were employed for diagnosis. Malaria diagnosis was done using a mRDT (SD Bioline, Standard Diagnostics, Inc., South Korea) and the procedures were performed according to the manufacturer’s instructions. From the same blood sample, thick and thin blood smears was prepared and stained with 3% Giemsa staining solution and examined under a light microscope with oil immersion for the presence of malaria parasite stages and quantification of malaria parasitaemia. A Giemsa-stained thin smear was used for the identification of malaria parasite species. Two laboratory technicians examined the slides independently and for quality assurance, 10% of the randomly selected positive and negative slides were re-examined by a third laboratory technician. Slides were considered negative for malaria infection if no parasites were seen in at least 200 oil immersion fields on the thick film. Malaria parasite density was determined according to the number of parasites per 200 white blood cells (WBCs), assuming a total WBC count of 8000/μL [19].

#### Estimation of haemoglobin levels

A sub-sample of the finger prick blood samples was used to determine the haemoglobin (Hb) level. Haemoglobin levels were measured using the Hemo-Control® system (EKF Diagnostic GmbH, Ebendorfer Chaussee 3, D-39179 Barleben/Magdeburg, Germany, www.EKF-diagnostic.com). Anaemia was defined as Hb < 11 g/dL for all study participants [20].

#### Screening for splenomegaly

Screening for splenomegaly was done to through palpation of the spleen when patient was lying on horizontal position by the research medical doctors and spleen enlargement was classified by Hackett’s methods [11]. Spleen examination was done in children ≤ 12 years old.

### Data analysis

Data were double entered into Epi Info™ version 6 (Center for Disease Control and Prevention, Atlanta, GA, USA) and then transferred to Stata® version 13 (StataCorp, College Station, TX, USA). Frequencies and percentages were used to describe the data. Continuous variables were described using median, interquartile range (IQR) and mean ± standard deviations. Categorical variables were described using the chi-square test (*χ* ^2^) or Fisher’s exact test where appropriate. Comparison of means or geometrical mean malaria parasite densities was done using t-tests for two variables and analysis of variance (ANOVA) for comparison of more than two variables. For logistic regression, factors with *P-*values of 0.2 were considered for the multivariable analysis. Factors such as sex, age in years, presence of anaemia and use of mosquito nets were considered for the multivariable analysis. Because of the interaction between splenomegaly and age, a splenomegaly variable was not considered for the multivariable analysis. The World Health Organization cut-off for Hb was used to classify anaemia [20]. A normal Hb level was defined as Hb ≥ 11 g/dL, mild anaemia was defined as Hb = 10.0–10.9 g/dL, moderate anaemia was defined as Hb = 7.0–9.9 g/dL, severe anaemia was defined as Hb = 4.0–6.9 g/dL and very severe anaemia was defined as Hb ≤ 3.99 g/dL [20].

### Ethical considerations

Ethical approval was obtained from the Catholic University of Health and Allied Sciences/Bugando Medical Centre and from the Medical Research Coordinating Committee of the National Institute for Medical Research, Tanzania. Permission to conduct this study was given by the Kilosa District Council Authority. Informed consent was obtained from adult individuals and for those who were < 18 years, an assent was obtained and informed consent was obtained from their parents/guardians on their behalf. Study participants detected with malaria parasites received antimalarial treatment from a qualified physician, as according to the Tanzania Malaria Control Guidelines for the Treatment and Management of Malaria.

## Results

### Demographic characteristics

A total of 1154 individuals participated in this study. Of these, 54.85% were females and 45.15% were males. Of all the study participants, 29.3% were primary school children aged 5–17 years. The mean age of the entire study cohort was 21.9 ± 19.69 years.

### Reported ownership and use of mosquito nets

A total of 86.7% of the study participants reported to own and use mosquito nets. There was a difference in terms of sex in relation to the reported use of mosquito nets, with female participants having a higher rate (55.9%) of using mosquito nets than males (44.1%) (*P* = 0.03). Study participants who reported not using mosquito nets had the highest prevalence of malaria parasites and had higher geometric mean parasite densities than those who reported using mosquito nets (19.91 parasites/μL versus 23.9 parasites/μL, *t* = 3.22, *P* = 0.001).

### Malaria prevalence and parasitaemia

*Plasmodium falciparum* accounted for the majority of the infections (89%), *P. malariae* accounted for 3.7% of the infections and 7.3% were mixed infections of *P. falciparum* and *P. malariae*. The overall prevalence of *P. falciparum* malaria infection was 14.2% based on microscopic examination and the geometric mean parasite density was 20.5 parasites/μL (95% *CI*: 14.6–28.8), with a marginal difference between the age groups (*F*-test = 2.13, *P* = 0.05) and significant differences between the sub-villages (*F*-test = 2.42, *P* = 0.03). Based on the mRDT results, the prevalence of *P. falciparum* infection was 17.5%. In general, 12.8% (*n* = 21/164) of the study participants infected with malaria parasites had gametocytes.

There was a significant difference in the prevalence of malaria between male and female participants, with males having a higher prevalence (*P* = 0.001). The age groups of 11–20 years (26.8%) and 1–10 years (22.5%) had the highest prevalence of malaria (see Table 1). On the other hand, based on the microscopic examination, malaria prevalence and parasitaemia was highest among individuals living in Mlegeni (23.9%) and Makuruwili (24.4%). However, there was no difference in the prevalence of malaria between age groups (see Table 1). Malaria prevalence varied significantly by sex (*P* = 0.011), sub-village of residence (*P* = 0.031) and reported use of mosquito nets (*P* = 0.004).

**Table 1:** Prevalence of malaria infection in relation to demographic and clinical characteristics based on malaria rapid diagnostic test and microscopic examination

Variables	Number	Malaria infection by malaria rapid diagnostic test				Number	Malaria infection by microscopy			
		Yes *n* (%)	No *n* (%)	Chi-square test	*P*-value		Yes *n* (%)	No *n* (%)	Chi-square test	*P*-value
Age groups (years)										
1–10	454	102 (22.5)	352 (77.5)	52.9345	0.001	456	62 (13.6)	394 (86.4)	1.8389	0.87
11–20	235	63 (26.8)	172 (73.2)			244	40 (16.4)	204 (83.6)		
21–30	139	10 (7.2)	129 (92.8)			140	22 (15.7)	118 (84.3)		
31–40	93	8 (8.6)	85 (91.4)			92	12 (13.0)	80 (86.9)		
41–51	88	8 (9.1)	80 (90.9)			89	11 (12.4)	78 (87.6)		
≥51	121	8 (6.2)	121 (93.8)			130	17 (13.1)	113 (86.9)		
Sex										
Female	626	92 (14.7)	534 (85.3)	7.5939	0.01	632	75 (11.9)	557 (88.1)	6.5054	0.011
Male	511	107 (20.9)	404 (79.1)			519	89 (17.2)	430 (82.9)		
Subvillages										
Chamwino	129	19 (14.7)	110 (85.3)	6.9861	0.22	129	19 (14.7)	110 (85.3)	12.2958	0.031
Kisiwani	122	23 (18.9)	99 (81.2)			123	18 (14.6)	105 (85.4)		
Kwamtunga	195	19 (9.7)	176 (90.3)			197	25 (12.7)	172 (87.2)		
Makuruwili	132	22 (16.7)	110 (83.3)			131	32 (24.4)	99 (75.6)		
Mlegeni	109	20 (18.4)	89 (81.7)			109	26 (23.9)	83 (76.2)		
Upogoroni	126	18 (14.3)	108 (85.7)			126	20 (15.9)	106 (84.1)		
Splenomegaly										
Absent	540	123 (22.8)	417 (77.22)	20.5162	0.001	551	70 (12.7)	481 (87.3)	4.0167	0.045
Present	17	12 (70.6)	5 (29.4)			17	5 (29.4)	12 (70.6)		
Anaemia (g/dl)										
Not anaemic	701	104 (14.8)	597 (85.2)	11.3177	0.001	707	87 (12.3)	620 (87.7)	6.3912	0.011
Anaemic	368	85 (23.1)	283 (76.9)			373	67 (17.9)	306 (82.0)		
Mosquito nets use										
No	153	33 (21.6)	120 (78.4)	1.9592	0.16	152	33 (21.7)	119 (78.3)	8.0835	0.004
Yes	980	166 (16.9)	814 (83.1)			995	130 (13.1)	865 (86.9)		

### Anaemia and splenomegaly

Only a total of 1083 out of 1154 study participants were screened for haemoglobin level because Hemo-cuvette for estimation of Hb levels using the Hemo-control system were only available for that number of study participants. About one-third (34.6%) of the study participants were anaemic (Hb < 11 g/dL). Of the anaemic participants, 4% (15/375), 49% (184/375) and 47% (176/375) were classified as having severe, moderate and mild anaemia, respectively. In relation to malaria infection, 3.5, 47.1 and 49.4% of the study participants with severe, moderate and mild anaemia were infected with malaria. The age groups of 1–10 years (48.6%) and 11–20 years (24.7%) had the highest prevalence of anaemia (*P* = 0.001). Study participants from Kisiwani (47.9%) and Makuruwili (42.4%) with anaemia had the highest prevalence of malaria (*P* = 0.001). Of the study participants with anaemia, 44.9% had malaria.

A total of 570 individuals were examined for spleen enlargement. Overall, the prevalence of enlarged spleen was 2.98% (17/570). All study participants who had splenomegaly were in the age groups of 1–10 years (2.1%) or 11–20 years (5.1%). Of the study participants with splenomegaly, 8.9% had malaria.

### Factors associated with malaria infection

In the bivariate analysis, malaria infection was mainly associated with being male, in the age group of 1–10 years or 11–20 years, having splenomegaly, and having moderate or mild anaemia (see Table 2). Use of mosquito nets was associated with a reduced odd of having malaria. However, the multivariable analysis showed that being in the age groups of 1–10 years (adjusted odds ratio, a*OR* = 4.41, 95% confidence interval, *CI*: 1.96–9.93, *P* = 0.001) or 11–20 years (a*OR* = 6.68, 95% *CI*: 2.91–15.37, *P* = 0.001), and having mild (a*OR* = 1.71, 95% *CI*: 1.11–2.62, *P* = 0.014) or moderate anaemia (a*OR* = 1.55, 95% *CI*: 1.01–2.39, *P* = 0.045) remained independently associated with malaria infection.

**Table 2:** Factors associated with malaria with malaria at Kilangali village, Kilosa district, central Tanzania

Variables	Response	c*OR*	95% *CI*	*P-*value	a*OR*	95% *CI*	*P*-value
Sex	Female	1			1		
	Male	1.53	1.13–2.09	0.01	1.31	0.94–1.82	0.11
Age (years)	1–10	4.38	2.07–9.27	0.01	4.41	1.96–9.93	0.001
	11–20	5.54	2.56–11.98	0.001	6.68	2.91–15.37	0.001
	21–30	1.17	0.44–3.06	0.75	1.37	0.48–3.84	0.55
	31–40	1.42	0.51–3.94	0.49	1.63	0.55–4.90	0.38
	41–50	1.52	0.54–4.19	0.43	1.88	0.65–5.46	0.24
	≥ 51	1					
Sub-villages	Chamwino	1					
	Kisiwani	1.35	0.69–2.62	0.38	----	-------	-------
	Kwamtunga	0.62	0.32–1.23	0.18	----	-------	-------
	Makuruwili	1.16	0.59–2.26	0.67	----	-------	-------
	Mlegeni	1.30	0.65–2.58	0.45	----	-------	-------
	Upogoroni	0.96	0.48–1.94	0.92	----	-------	-------
Splenomegaly	No	1					
	Yes	8.14	2.81–23.54	0.001	----	-------	-------
Anaemia (g/dl)	10.0–10.9	1.88	1.25–2.83	0.001	1.71	1.11–2.62	0.014
	7.0–9.9	1.61	1.07–2.41	0.002	1.55	1.01–2.39	0.045
	4.0–6.0	1.44	0.39–5.17	0.023	1.43	0.38–5.35	0.59
Net use	No	1					
	Yes	0.54	0.34–0.83	0.005	0.69	0.44–1.12	0.14

## Discussion

The findings of the present study indicate that there is malaria infection in the Kilangali village and that *P. falciparum* accounts for the majority of the cases. In Tanzania, *P. falciparum* accounts for more than 95% of the malaria cases reported in health facilities [13]. Similarly, in rice farming communities in Tanzania and elsewhere, where malaria has been noted as a serious public health problem, *P. falciparum* parasites have been repeatedly observed to be the leading cause of symptomatic and asymptomatic malaria infections and disease [8, 9, 11, 21, 22]. Flooded rice farming practice is known to provide suitable breeding sites for the malaria vectors, *Anopheles gambiae*, in Africa [9]. This ensures the continual transmission of malaria in rice farming communities.

The prevalence of *P. falciparum* malaria observed varied significantly according to age and sex, with the youngest age groups having a higher prevalence of malaria. Recent studies in the same district [21, 22] and neighbouring district of Mvomero [11] have observed high malaria prevalence and parasitaemia among school-age children [21]. However, some studies have also reported the lowest malaria prevalence among rice irrigation communities in Sub-Saharan Africa [23, 24]. The observed variation in malaria prevalence observed could partly be explained by variation in micro-ecological factors such as the type of rice farming (flooded farming) practices and distribution of malaria vectors breeding sites, as well as socio-economic factors [9].

In this study, males had a higher prevalence of malaria infection than females. This is mostly likely to be a reflection of the variation in behaviour related to exposure [25]. Our own data and reports from other authors indicate that women are more likely to use mosquito nets than men [25], and this may partly explain the difference in malaria prevalence. Mosquito nets have been demonstrated to reduce the risk of acquiring malaria infection among individuals who report using mosquito nets [21, 26]. However, other community studies have not observed a difference relating to sex in terms of malaria prevalence [21].

As expected, our findings have demonstrated a significant difference in malaria prevalence and parasitaemia between the sub-villages. Prevalence of *P. falciparum* malaria was higher among inhabitants of Makuruwili and Mlegeni, which are located close to a large commercial rice seed irrigation scheme. Other authors have demonstrated higher malaria prevalence in inhabitants living in households located close to traditional flooded rice irrigation schemes [11, 22]. Significant variations in the level of parasitaemia between villages within the rice irrigation agrosystem have been noted [22]. The risk of malaria transmission have been noted to vary even on the smallest scale [27] and in part can be explained by variations in socio-demographic and socio-economic factors.

Anaemia was also prevalent among the study participants and the youngest age group had the highest prevalence. In addition, the prevalence of anaemia varied significantly by village of residence, with study participants from the sub-villages of Kisiwani and Makuruwili having the highest prevalence of anaemia. Previous studies have recorded high prevalence of anaemia among school-age children from rice farming communities in the Kilosa [21, 22] and Mvomero districts [28]. High prevalence of anaemia (62.6%) among school-age children has been noted elsewhere in Tanzania [29, 30]. In the present study, slightly below half of the study participants with anaemia had malaria infection. Pathogenically, malaria parasites are responsible for causing anaemia [31, 32]. However, anaemia has multiple causes including nutrition deficiencies and parasitic infections other than malaria [28–30]. In general, anaemia remains one of the serious public health problems among children and pregnant women in Tanzania [33], with the major contributing factor being inadequate dietary intake of nutrients [34].

As shown in previous studies [30, 32, 35], malaria infection was mainly associated with anaemia and young age groups. Malaria parasites are responsible for causing anaemia among school children in Sub-Saharan Africa [30, 35, 36]. However, it is worthwhile to note that anaemia in the region has multiple causes that were not evaluated in the present study. Nevertheless, the age distribution of malaria prevalence, parasitaemia and intensity of infection observed in the present study indirectly suggest that the study setting is characterised by a stable malaria transmission [37] and development of malaria-related acquired immunity [38–40]. Malaria prevalence and parasite density among infected study participants were significantly higher in the age groups of 1–10 years and 11–20 years. Adults aged > 20 years had the lowest malaria prevalence and malaria parasite density. These observations clearly indicate that children are exposed to malaria parasites at early ages because of high transmission and develop malaria-related immunity as they grow older [40]. Contrasting observations have been reported in the Bioko island in Equatorial Guinea, where children aged less than five years had the lowest malaria prevalence and parasitaemia, indicating the age group was less exposed [41]. Risk factors associated with malaria infection vary from one transmission setting to another, and are mainly influenced by socio-economic and ecological factors.

The present study was not conducted without limitations. The use of expert microscopy and rapid diagnostic tests may have missed light infection when compared to using sensitive molecular methods such as polymerase chain reactions [42]. In addition, the present study did not collect household information, which is important for assessing the risk factors associated with malaria. Lastly, the cross-sectional nature of the study may have led to a lack of temporal association between malaria and other study variables.

## Conclusion

Our results indicate that there is malaria infection in the study setting and that the prevalence varies according to age group, sex, sub-village of residence and reported use of mosquito nets. Further studies are needed to understand the observed variation in malaria prevalence and contribution of household and socio-economic factors.

## Additional file

Additional file 1: Multilingual abstracts in the five official working languages of the United Nations. (PDF 751 kb)

## Abbreviations

*aOR*: Adjusted odds ratio
*CI*: Confidence interval
Hb: Haemoglobin
IQR: Interquartile range
mRDT: Malaria rapid diagnostic test
WBC: White blood cell
