# Supplementary material for: Malaria among rice farming communities in Kilangali village, Kilosa district, Central Tanzania: prevalence, intensity and associated factors
Source: Infect Dis Poverty. 2017 Jul 5;6:101. doi: 10.1186/s40249-017-0315-1 (PMC5497374; doi:10.1186/s40249-017-0315-1)
Supplement: Additional file 1: — Multilingual abstracts in the five official working languages of the United Nations. (PDF 751 kb) [file 40249_2017_315_MOESM1_ESM.pdf]

Translation of the abstract into the five official working languages of the United Nations

الملاريا بين مجتمعات زراعة الأرز في قرية كيلانجالي، إقليم كيلوسا، وسط تنزانيا: الانتشار والكثافة وما يرتبط بهما من عوامل

همفري د. مازيجو، سوزان ف روميشا، ميرسي ج. تشيدو، فينيراندا م بوانا، ليونارد إ. ج. مبويرا

ملخص

خلفية: لا تزال الملاريا أهم أسباب المراضة والوفيات في تنزانيا. ومع ذلك، انتشارها يختلف من منطقة إلى أخرى اعتمادا على مختلف العوامل البيئية والاجتماعية والاقتصادية والنظام الصحي. وقد أجريت هذه الدراسة لتحديد انتشار الملاريا والعوامل المرتبطة بها بين مجتمعات زراعة الأرز في قرية كيلانجالي في منطقة كيلوسا في تنزانيا الوسطى.

أساليب: وقد أجريت دراسة مستعرضة في مايو 2015، وشملت أشخاصا تم اختيارهم عشوائيا يعيشون في ست قرى تابعة لقرية كيلانجالي، وهي ملينجيني، كيسيواني، ماكوروويلي، كوامتونجا، أوبوجوروني، تشاموينو. تم الحصول على عينة من الدم بوخزالإصبع لتشخيص الإصابة بالملاريا باستخدام مسحات سميكة ملطخة بالغيمزا واختبار الملاريا التشخيصي السريع. تم فحص المشاركين في الدراسة أيضا لبيان مستويات الهيموجلوبين وتم فحص ما مجموعه 570 طفلا تتراوح أعمارهم بين ≤ 12 سنة من العمر لمعرفة المصابين بتضخم الطحال باستخدام طريقة الجس.

النتائج: تم فحص ما مجموعه 1154 شخصا لعدوى الملاريا بمتوسط عمر 21.9 ± 19.69 عاما. وكان انتشار الملاريا الإجمالي 14.2٪ و 17.5٪ على أساس الفحص المجهري والاختبار التشخيصي السريع، على التوالي. المتصورة المنجلية تستأثر بأغلبية (89٪) من الإصابات بمرض الملاريا. كان الوسط الهندسي الإجمالي لكثافة الطفيليات 20.5 طفيلي / ميكرولتر (95٪ CI: 14،6- 28،8). كان انتشار الملاريا والطفيليات في الدم أعلى بين الأفراد الذين يعيشون في القريتين التابعتين مليجيني(23.9٪) وماكوروويلي (24.4٪). ومن بين الأطفال الذين فصحوا للإصابة تضخم الطحال، كان 2.98٪ (570/17) مصابين. وكان معدل انتشار فقر الدم 34.6٪.

الاستنتاجات: تم العثور على الملاريا أثناء إعداد الدراسة وانتشاره اختلف وفقا للخصائص الديموجرافية للمشاركين في الدراسة وبين القرى التابعة التي شديدة القرب.

Translated from English version into Arabic by Mahmoud Sami, through

TRANSLATORS
WITHOUT BORDERS

坦桑尼亚中部 Kilosa 区 Kilangali 村水稻农业社区疟疾患病率、流行强度及其相关因素

Humphrey D. Mazigo, Susan F. Rumisha, Mercy G. Chiduo, Veneranda M. Bwana, Leonard E. G. Mboera

摘要

引言：在坦桑尼亚，疟疾仍是导致疾病和死亡的主要疾病之一。然而，生态环境、社会经济和卫生体系等因素的差异导致在不同地区该病的流行率不同。本研究目的在于确定坦桑尼亚中部Kilosa区Kilangali村水稻农业社区疟疾患病率、流行强度及其相关因素。

方法：于2015年5月开展一项横断面研究，随机选取Kilangali村6个下级村村民进行研究，分别为Mlegeni、Kisiwani、Makuruwili、Kwamtunga、Upogoroni和Chamwino。通过指尖采血，采用吉氏染色厚血涂片和快速诊断试剂进行诊断。同时测定其血红蛋白水平，对570名≤ 12岁的儿童采用扪诊进行脾脏肿大检查。

结果：共计对154名村民进行了疟疾感染检测，平均年龄为21.9±19.7岁。镜检和快速诊断试剂确认的疟疾患病率分别为14.2%和17.5%。以恶性疟为主，占89%。原虫密度几何平均数为20.5 parasites/µl(95% *CI*: 14.6–28.8)。疟疾流行率和原虫血症在Mlegeni (23.9%)和Makuruwili (24.4%)最高。发现2.98% (17/570)的儿童出现脾脏肿大。贫血的患病率为34.6%。疟疾感染与年龄组1–10岁 (a*OR*=4.41, 95% *CI*: 1.96–9.93, *P*<0.001)和11–20岁(a*OR*=6.68, 95% *CI*: 2.91–15.37, *P*<0.001) 相关，且与轻度贫血 (a*OR*=1.71, 95% *CI*: 1.11–2.62, *P*<0.014) and 中度贫血 (a*OR*=1.55, 95% *CI*: 1.01–2.39, *P*<0.045) 相关。
结论：本研究在研究区域发现了疟疾，并发现患病率随参与人员人口特征变化，在距离很近的下级村患病率也存在差异。

Translated from English version into Chinese by Xin-Yu Feng, edited by Yang Pin, through

CHINESE CENTER FOR DISEASE CONTROL AND PREVENTION
NATIONAL INSTITUTE OF PARASITIC DISEASES

Présence du paludisme parmi des communautés rizicoles dans le village de Kilangali, district de Kilosa, Tanzanie centrale : prévalence, intensité et facteurs associés

Humphrey D. Mazigo, Susan F. Rumisha, Mercy G. Chiduo, Veneranda M. Bwana, Leonard E. G. Mboera

RÉSUMÉ

Contexte : le paludisme reste la principale cause de morbidité et de mortalité en Tanzanie. Sa prévalence varie néanmoins en fonction des régions et dépend de nombreux facteurs écologiques et socio-économiques ainsi que de facteurs liés au système de santé. La présente étude a été menée afin de déterminer la prévalence du paludisme et des facteurs associés parmi des communautés rizicoles du village de Kilangali, district de Kilosa, Tanzanie centrale.
Méthodes : une étude transversale a été menée en mai 2015 auprès de personnes sélectionnées de manière aléatoire dans les six sous-villages du village de Kilangali : Mlegeni, Kisiwani, Makuruwili, Kwamtunga, Upogoroni et Chamwino. Un échantillon de sang prélevé à l'extrémité du sang a été recueilli à des fins de diagnostic du paludisme à l'aide de la coloration de Giemsa de gouttes épaisses et d'un test de diagnostic rapide du paludisme. Les participants à l'étude ont aussi fait l'objet d'un examen de mesure des taux d'hémoglobine et un total de 570 enfants âgés de ≤ 12 ans ont subi un examen de dépistage d'une hypertrophie de la rate à l'aide de la méthode par palpation.
Résultats : au total, 1154 personnes ont fait l'objet d'un examen de dépistage du paludisme, leur âge moyen s'élevait à 21,9 ± 19,69 ans. Le taux de prévalence global du paludisme s'élevait à respectivement 14,2 % et à 17,5 % pour l'examen par microscope et le test de diagnostic rapide. Le *Plasmodium falciparum* était responsable de la majorité (89 %) des cas de paludisme. La moyenne géométrique globale de la densité parasitaire était de 20,5 parasites/µL (*IC* à 95 % : 4,6 – 28,8). La prévalence du paludisme et la parasitémie étaient les plus élevées chez les individus vivant dans les sous-villages de Mlegeni (23,9 %) et Makuruwili (24,4 %). Parmi les enfants ayant fait l'objet d'un examen de dépistage d'une splénomégalie, 2,98 % (17/570) avaient obtenu un résultat positif. La prévalence globale de l'anémie s'élevait à 34,6 %. L'infection paludique était associée aux groupes d'âge de 1 à 10 ans (a*OR* = 4,41, *IC* à 95 % : 1,96 – 9,93, *P* < 0,001) et de 11 à 20 ans (a*OR* = 6,68,

IC à 95 % : 2,91 – 15,37, *P* < 0,001) ainsi qu'à une anémie légère (a*OR* = 1,71, *IC* à 95 % : 1,11–2,62, *P* < 0,014) et une anémie modérée (a*OR* = 1,55, *IC* à 95 % : 1,01 – 2,39, *P* < 0,045).
Conclusions : le paludisme a été identifié sur le site d'étude et sa prévalence affichait des variations conformes aux caractéristiques démographiques des participants à l'étude et parmi les sous-villages proches.

Translated from English version into French by eric ragu, through

Малярия среди рисовыращивающих фермерских общин в деревне Килангали района Килоса в Центральной Танзании: Распространенность, плотность и связанные с ними факторы

Хэмфри Д. Мазиго, Сьюзан Ф. Румиша, Мерси Г. Чидуо, Венеранда М. Бвана, Леонард Е. Г. Мбоера

РЕФЕРАТ

Предпосылки: Малярия остается наиболее важной причиной заболеваемости и смертности в Танзании. Однако, ее распространенность варьирует от территории к территории в зависимости от различных экологических, социально-экономических факторов и факторов системы здравоохранения. Настоящее исследование проводилось с целью определить распространенность малярии и связанные с этим факторы среди рисовыращивающих фермерских общин в деревне Килангали района Килоса в Центральной Танзании.
Методы: В мае 2015 года было проведено перекрестное исследование, включавшее случайно выбранных людей, проживающих в шести суб-деревнях деревни Килангали, а именно: Мленги, Кисивани, Макурувили, Квамтунга, Упогорони и Чамвино. Из прокола пальца бралась проба крови для диагностики малярийной инфекции с помощью густых мазков красителя Гимза и быстрого теста для диагностики малярии. Проводился также скрининг участников исследования на уровни гемоглобина, а у 570 детей возрасте не старше 12 лет методом пальпации определялось увеличение селезенки.
Результаты: Всего на малярийную инфекцию было обследовано 1154 людей в среднем возрасте 21,9 ± 19,69 лет. Общая распространенность малярии, определенная на основании микроскопического исследования и быстрого диагностического теста была, соответственно, 14,2% и 17,5 %. Большинство случаев заражения малярией (89%) относилось на счет *Plasmodium falciparum.* Общая средняя геометрическая плотность паразитов была 20,5 паразитов/мкл (в доверительном интервале 95%: 14,6 ... 28,8). Распространенность и паразитемия были наивысшими у проживающих в суб-деревнях Мленги (23,9%) и Макурувили (24,4%). У детей проверялась спленомегалия, ее имели 2,98% (17/570). Общая распространенность анемии была 34,6%. Малярийная инфекция была связана с возрастными группами 1 … 10 лет (a*OR* = 4,41, в доверительном интервале 95%: 1,96 – 9,93, *P* < 0,001) и

11 ... 20 лет (a*OR* = 6,68, в доверительном интервале 95%: 2,91 – 15,37, *P* < 0,001) и слабой анемией (a*OR* = 1,71, в доверительном интервале 95%: 1,11 – -2,62, *P* < 0,014) и умеренной анемией (a*OR* = 1,55, в доверительном интервале 95%: 1,01 – 2,39, *P* < 0,045).
Выводы: В исследовании обнаружено, что обнаруженная малярия и ее распространенность варьируют в зависимости от демографических характеристик участников исследования и между близко расположенными суб-деревнями.

Translated from English version into French by Alexander Somin, through

TRANSLATORS
WITHOUT BORDERS

Malaria en comunidades de cultivadores de arroz en el pueblo de Kilangali, distrito de Kilosa, Tanzania Central: Predominio, intensidad y factores asociados

Humphrey D. Mazigo, Susan F. Rumisha, Mercy G. Chiduo, Veneranda M. Bwana, Leonard E. G. Mboera

RESUMEN

Antecedentes: La malaria continúa siendo la causa más importante de morbilidad y mortaidad en Tanzania. No obstante, su predominio varía de zona a zona según factores varios, como ecológicos, socioeconómicos y de sistemas de salud. Este estudio se llevó a cabo para determinar el predominio de la malario y los factores asociados entre las comunidades de cultivadores de arroz en el pueblo de Kingali, del distrito de Kilosa en Tanzania Central.
Métodos: Se efectuó un estudio transversal en Mayo de 2015, en el que se seleccionaron al azar personas que vivían en los seis subpoblados del pueblo de Kingaly, a saber, Mlegeni, Kisiwani, Makuruwili, Kwamtunga, Upogoroni y Chamwino. Se sacó una muestra de una gota de sangre para diagnosticar la infección de malaria mediante gotas gruesas teñidas por Giemsa y una prueba rápida de diagnóstico de malaria. También se examinaron a los participantes del estudio para ver los niveles de hemoglobina y se examinaron un total de 570 niños menores de 12 años para observar engrandecimiento del bazo, mediante el método de palpación.
Resultados: Se examinó un total de 1.154 personas para observar si existía infección de malaria, con una edad promedio de 21.9 ± 19.69 años. El predominio total de malaria fue de 14.2% y 17.5% , según un examen microscópico y una prueba ráidad diagnóstica, respectivamente. *Plasmodium falciparum* era el causante de la mayoría (89%) de infecciones de malaria. La densidad geométrica principal global era de 20.5 parasitos/µL (95% *CI*: 14.6 – 28.8). El predominio de malaria y parasitemia era mayor entre los individuos que vivían en los subpoblados de Mlegeni (23.9%) y Makuruwili (24.4%) . Entre los niños observados para ver si sufrían esplenomegalia, 2.98% (17/570) la sufrían. El predominio total de anemia era de 34.6%. La infección de malaria se asociaba a grupos de edad de 1 – 10 años (a*OR* = 4.41, 95% *CI*: 1.96 – 9.93, *P* < 0.001) y 11 – 20 años (a*OR* = 6.68, 95% *CI*: 2.91 – 15.37, *P* < 0.001); y la anemia ligera (a*OR* = 1.71, 95% *CI*: 1.11–2.62, *P* < 0.014) y moderada (a*OR* = 1.55, 95% *CI*: 1.01 – 2.39, *P* < 0.045).

Conclusiones: Se observó la malaria en el ámbito del estudio y su predominio variaba según las características demográficas de los participantes del estudio y entre los subpoblados que se encuentran cerca unos de otros.

Translated from English version into Spanish by ISABEL SANLLEHI, through

TRANSLATORS
WITHOUT BORDERS
